# Analysis of the utilization and effectiveness of tuberculosis digital adherence technology in southeastern China

**DOI:** 10.3389/fpubh.2026.1759922

**Published:** 2026-01-16

**Authors:** Daiquan Chen, Zhisong Dai, Shufang Lin, Shuzhen Wei, Kun Chen, Yanqin Deng

**Affiliations:** 1 Fujian Center for Disease Control and Prevention, Fuzhou, Fujian, China; 2 Fujian Provincial Key Laboratory of Zoonosis Research, Fuzhou, Fujian, China

**Keywords:** community management, digital adherence technologies, epidemiologic factors, treatment adherence, tuberculosis

## Abstract

**Background:**

In response to the World Health Organization’s advocacy for patient-centered care, Fujian Province, China, initiated the promotion of Digital Adherence Technology (DAT) in 2024 as an optional strategy to enhance treatment adherence—a critical determinant of patient outcomes. This study aimed to evaluate the real-world implementation and effectiveness of this program.

**Methods:**

We conducted a multi-site study in four prefecture-level cities where DAT had been deployed to over 100 patients. Data were extracted from the national Tuberculosis Information Management System and linked with DAT platform records. Patients with DAT records were assigned to the DAT group, others to the conventional care group. We analyzed factors associated with DAT non-adoption. Adherence was compared between groups using three indicators: sputum smear examination rate at month two, smear conversion rate at month two (among bacteriologically confirmed patients), and loss to follow-up rate. Propensity score matching (PSM) was employed to balance baseline characteristics, followed by subgroup analyses.

**Results:**

The overall DAT utilization rate was 50.90%. Protective factors against DAT non-use included female sex (aOR = 0.829, 95% CI: 0.716–0.960), non-Han ethnicity (aOR = 0.555, 95% CI: 0.343–0.898), use of fixed-dose combination therapy (aOR = 0.806, 95% CI: 0.666–0.975), and concurrent extrapulmonary TB (aOR = 0.761, 95% CI: 0.642–0.902). Compared to Fuzhou, patients in Putian, Quanzhou, and Ningde had significantly lower odds of non-use (aORs: 0.024, 0.076, 0.030). Migrant status (aOR = 2.621, 95% CI: 2.144–3.203) and symptomatic presentation at diagnosis (aOR = 1.604, 95% CI: 1.401–1.836) were risk factors. The DAT group demonstrated a significantly higher month-two smear examination rate than the conventional group in both the full and PSM-matched cohorts (*p* < 0.001). No significant inter-group differences were found in month-two smear conversion rates or loss to follow-up.

**Conclusion:**

DAT utilization in Fujian remains moderate and is influenced by specific demographic and clinical factors. While DAT can improve process adherence indicators, its impact on definitive treatment outcomes requires further investigation. Optimization of patient management should include formal integration of DAT, workflow streamlining, and personalized intervention strategies. Future research must develop and evaluate effective interventions for DAT-identified non-adherent patients to translate monitoring data into improved clinical outcomes.

## 1 Introduction

Tuberculosis (TB) remains a major global public health challenge. In 2024, TB caused an estimated 10.7 million new cases and 1.23 million deaths, retaining its position as the leading cause of mortality from a single infectious agent. Although China is now classified as a low-to-middle incidence country (49 cases per 100,000 population), it continues to bear a substantial disease burden. In 2024, China reported 696,000 new TB cases, accounting for 6.5% of the global total and ranking fourth worldwide in terms of total case numbers (1).

Treatment non-adherence profoundly influences TB outcomes (2). Since 2005, the standard of care for TB in China has been Directly Observed Treatment (DOT), a strategy where medication intake is supervised by a healthcare worker or trained observer to enhance adherence (3). Under the National Tuberculosis Control Program (NTP), all registered patients are managed using DOT to ensure compliance, contributing to a 95% treatment success rate for new and relapsed drug-sensitive TB in 2024 (1). However, the implementation of DOT presents significant challenges for patients, exacerbated by factors such as stigma, solitary living, frequent travel, and geographical barriers—the latter being particularly problematic in remote rural areas. A systematic review indicated that only 20% of patients received therapy observed by a health professional, while over 50% self-administered their treatment (4). Applying DOT uniformly to all patients is also inconsistent with the people-centered care approach advocated by the WHO consolidated guidelines (5). However, it should be noted that current Chinese TB patient management guidelines do not formally incorporate DATs as a standard component of patient care.

Accumulating evidence from global and Chinese studies suggests that widely implemented digital adherence technologies (DATs) can effectively address these challenges while demonstrating efficacy comparable to that of DOT. Multicenter randomized controlled trials in Tibet (China) and England have shown that DATs can achieve better treatment adherence than DOT (6, 7). Several studies have confirmed the effectiveness of DATs in improving adherence (3, 8), with some demonstrating non-inferiority to DOT (9, 10). A cluster-randomized trial across four countries found no significant difference in unfavorable outcomes between DAT and control groups (11). Furthermore, research indicates that DATs can substantially reduce treatment costs for both patients and healthcare systems (12, 13).

Despite these promising findings, current research has predominantly focused on the effectiveness, acceptability, and feasibility of DATs, with limited investigation into their real-world uptake rates and determining factors. For instance, one study reported that 35.4% of patients pre-allocated to a DAT group did not use the intervention (11). An observational study from China found that nearly 30% of patients declined DAT, with specific groups such as older adults showing significantly lower acceptance (14). Interestingly, other studies, such as that by Kevin Guzman and colleagues, have reported greater acceptance of electronic adherence tools among older adults compared to younger individuals (15), highlighting the need for context-specific understanding.

Beginning in 2024, Fujian Province initiated a province-wide implementation of digital adherence technology. The adoption of this intervention follows a shared decision-making process between patients and clinicians at designated hospitals. Currently, there is a lack of data on the utilization, effectiveness, and factors influencing the non-adoption of DATs in Fujian Province. This study aims to evaluate the real-world implementation and the effectiveness of DATs in improving treatment adherence. The findings will provide critical evidence to guide the strategic implementation of digital adherence technologies in Fujian and similar settings.

## 2 Method

### 2.1 Study design and setting

This cross-sectional study was conducted in Fujian Province, located in southeastern China, which had a resident population of 41.93 million in 2024. In the same year, the province reported over 13,000 new pulmonary tuberculosis (TB) cases, corresponding to an incidence rate of 34.6 per 100,000 population. Since June 2024, the province has implemented a province-wide rollout of the Yi Du Dao digital adherence technology (DAT) platform, developed by Beijing SINOVO Power Technology Company (China). This initiative provides two options for patients: a smart pillbox with reminder functions and automatic adherence data syncing, or a mobile health (mHealth) intervention utilizing WeChat for reminders and self-reporting of medication intake. All designated healthcare workers across the province received standardized training on the use of these technologies prior to implementation. While all patients continued to receive standard supervision under the National Tuberculosis Control Program (NTP), they were also offered the optional use of either DAT tool as an adherence support adjunct, which was administered by healthcare workers in the designated hospital. For this analysis, patients who received only standard NTP supervision were classified as the Routine Care group, whereas those who opted to supplement NTP supervision with either DAT tool were classified as the DAT group. As DAT implementation was not uniform across all prefectures, the analysis focused on the four prefecture-level cities (Fuzhou, Putian, Quanzhou, and Ningde) where the number of DAT users exceeded 100. All drug-susceptible TB patients residing in the four prefecture-level cities were included in the study.

### 2.2 Data sources and participant classification

The study included all patients with drug-susceptible tuberculosis (DS-TB), defined as bacteriologically confirmed or clinically diagnosed cases without evidence of rifampicin resistance, who were registered between July 2024 and June 2025. Exclusion criteria comprised: (1) baseline rifampicin resistance confirmed by drug susceptibility testing; (2) diagnosis of exclusively extrapulmonary TB; (3) registration without subsequent initiation of anti-TB therapy; (4) laboratory-confirmed non-tuberculous mycobacterial infection during the treatment course; and (5) a final treatment outcome of “diagnosis changed.” Patient data were extracted from two primary sources: (1) the Tuberculosis Information Management System (TBIMS) of the Chinese Center for Disease Control and Prevention, which included all DS-TB patients registered for treatment from July 2024 to June 2025; and (2) the DAT platform, which logged records of patients who used the adherence technologies. The cohort was constructed by cross-referencing the list of DS-TB patients from the TBIMS with the DAT platform records. Patients with a matching record on the DAT platform were assigned to the DAT group; those without a matching record were assigned to the Routine Care group. With reference to the study by Wang et al. (14), patients were stratified by age into two groups (≥65 and <65 years). According to the results of sputum smear, culture, or molecular testing at diagnosis, patients were defined as bacteriologically positive if any test was positive, and bacteriologically negative otherwise. Co-existing TB was defined as pulmonary tuberculosis concurrent with extrapulmonary tuberculosis.

### 2.3 Assessment of treatment adherence

Due to the unavailability of treatment adherence monitoring data for patients in Routine group, treatment adherence assessment relied solely on data from the TBIMS. Since the majority of enrolled patients had not yet completed their minimum 6-month treatment regimen by the data extraction date (September 1, 2025), we therefore utilized the following surrogate indicators to evaluate the treatment adherence: 1 completion of sputum smear microscopy at the end of the second treatment month, 2 sputum smear conversion rate among bacteriologically confirmed patients at the second month, and 3 overall loss to follow-up rate. A patient was considered to have completed the month-2 smear test if a test result was documented in the TBIMS, irrespective of the result. Among patients with bacteriologically confirmed pulmonary TB at diagnosis, smear conversion at the second month was defined as a negative smear result; otherwise, the case was classified as non-converted. Loss to follow-up was defined based on the treatment outcome documented in the TBIMS by the data extraction date. Patients whose outcomes were recorded as “Lost to Follow-up” or “Other” were classified as lost to follow-up, and the rate was calculated as the proportion of such patients in the total cohort.

### 2.4 Statistical analysis

All analyses were performed using R software (version 4.4.3). Continuous variables with normal distribution were expressed as mean ± standard deviation and compared using the independent samples t-test. Categorical variables were summarized as frequencies and percentages, with group differences assessed using the chi-square test. To identify factors associated with the uptake of DATs, multivariate logistic regression was employed. To evaluate the impact of DAT on the adherence outcomes, propensity score matching (PSM) was conducted in a 1:1 ratio between the DAT and Routine Care groups. The matching used the nearest neighbor method with a caliper width of 0.0001 to minimize the propensity score distance between matched pairs. A two-sided *p*-value < 0.05 was considered statistically significant for all tests.

## 3 Results

### 3.1 Demographic and clinical characteristics

From July 2024 to June 2025, a total of 7,626 patients with drug-susceptible tuberculosis were registered across the four participating prefectural cities of Fujian Province. Among them, 5,773 were male and 1,853 were female, with a mean age of 54.94 ± 16.90 years. Of these, 5,146 were under 65 years old, and 2,480 were aged 65 or older. A total of 3,882 patients received DAT as an intervention to support treatment adherence, representing a DAT utilization rate of 50.90% (Table 1).

**Table 1:** Differences in DAT utilization rates across patient subgroups.

Characteristics	Total	Use DAT	Utilization rate of DAT (%)	*χ^2^*	*p*-value
Overall	7,626	3,882	50.90		
Gender				0.005	0.946
Male	5,773	2,940	50.93		
Female	1853	942	50.84		
Age (years)				1.800	0.180
<65	5,146	2,647	51.44		
≥65	2,480	1,235	49.80		
Ethnic				22.248	0.000
Han	7,499	3,791	50.55		
Other ethnicities	127	91	71.65		
Occupation				213.385	0.000
Employed in enterprises or public institutions	165	57	34.55		
Students or children	261	133	50.96		
Worked in public places or commercial services	182	98	53.85		
Manual laborers	3,757	2,215	58.96		
Retired/Unemployed	2,947	1,239	42.04		
Other occupations	314	140	44.59		
Specific populations				0.003	0.959
No	5,253	2,673	50.89		
Yes	2,373	1,209	50.95		
Residence				1119.244	0.000
Local	5,909	3,618	61.23		
Migrant	1717	264	15.38		
City of residence				2887.662	0.000
Fuzhou	2,582	240	9.30		
Putian	995	844	84.82		
Quanzhou	3,149	2041	64.81		
Ningde	900	757	84.11		
Symptom status				239.823	0.000
Asymptomatic	2,298	1,480	64.40		
Symptomatic	5,328	2,402	45.08		
Case finding				24.186	0.000
Active	90	69	76.67		
Passive	7,536	3,813	50.60		
TB cases classification				7.991	0.005
Positive	5,422	2,816	51.94		
Negative	2,204	1,066	48.37		
Comorbidities				9.434	0.002
No	5,317	2,645	49.75		
Yes	2,309	1,237	53.57		
TB type				7.421	0.006
New cases	7,093	3,641	51.33		
Previously treated cases	533	241	45.22		
Use of FDC				742.404	0.000
No	1,677	361	21.53		
Yes	5,949	3,521	59.19		
Co-existing TB				1.124	0.289
No	6,540	3,313	50.66		
Yes	1,086	569	52.39		

### 3.2 Factors influencing DAT use in patients with DS-TB

Univariable logistic regression analysis identified that other ethnicities, the presence of comorbidities, the use of FDC, and co-existing extrapulmonary tuberculosis were significant protective factors against the use of DAT, with crude odds ratios (cORs) of 0.404 (0.274–0.597), 0.858 (0.778–0.946), 0.189 (0.167–0.215), and 0.763 (0.685–0.851) respectively. Compared to patients employed in enterprises or public institutions, those who were students or children, worked in public places or commercial services, were manual laborers, or had other occupations had significantly lower odds of not using DAT, with crude odds ratios (cORs) of 0.508 (0.340–0.760), 0.452 (0.293–0.698), 0.367 (0.265–0.510), and 0.656 (0.444–0.969), respectively. Compared with Fuzhou, patients in Putian, Quanzhou, and Ningde were significantly less likely to be non-users of DAT, with cORs of 0.018 (0.015–0.023), 0.056 (0.048–0.065), and 0.019 (0.015–0.024), respectively. The factors associated with increased odds of DAT non-use were migrant status (OR = 8.692; 95% CI: 7.547–10.010), passive case finding (OR = 1.836; 95% CI: 1.340–2.516), presenting with TB-related symptoms at diagnosis (OR = 2.204; 95% CI: 1.992–2.438), bacteriologically negative results (OR = 3.208; 95% CI: 1.964–5.240), and a history of previously treated TB (OR = 1.278; 95% CI: 1.071–1.525) (Table 2).

**Table 2:** Logistic regression analysis of factors influencing DAT utilization.

Characteristics	Univariate analysis		Multivariate analysis	
	Crude OR (95%CI)	*P*-value	Adjust OR (95%CI)	*P*-value
Gender				
Male	1		1	
Female	1.004 (0.904–1.114)	0.946	0.829 (0.716–0.960)	0.012
Ethnic				
Han			1	
Other ethnicities	0.404 (0.274–0.597)	<0.001	0.555 (0.343–0.898)	0.016
Occupation				
Employed in enterprises or public institutions	1		1	
Students or children	0.508 (0.340–0.760)	0.001	0.793 (0.446–1.410)	0.430
Worked in public places or commercial services	0.452 (0.293–0.698)	<0.001	0.721 (0.400–1.303)	0.279
Manual laborers	0.367 (0.265–0.510)	<0.001	1.004 (0.627–1.606)	0.987
Retired / Unemployed	0.728 (0.524–1.011)	0.058	0.722 (0.449–1.159)	0.177
Other occupations	0.656 (0.444–0.969)	0.034	1.308 (0.758–2.257)	0.335
Age (years)				
<65	1		1	
≥65	1.068 (0.970–1.175)	0.180	0.921 (0.809–1.049)	0.213
Specific populations				
No	1		1	
Yes	0.997 (0.905–1.099)	0.959	1.219 (0.944–1.573)	0.130
Residence				
Local	1		1	
Migrant	8.692 (7.547–10.010)	<0.001	2.621 (2.144–3.203)	<0.001
City of residence				
Fuzhou				
Putian	0.018 (0.015–0.023)	<0.001	0.024 (0.018–0.030)	<0.001
Quanzhou	0.056 (0.048–0.065)	<0.001	0.076 (0.063–0.092)	<0.001
Ningde	0.019 (0.015–0.024)	<0.001	0.030 (0.024–0.039)	<0.001
Case finding				
Active	1		1	
Passive	1.836 (1.340–2.516)	<0.001	1.133 (0.657–1.955)	0.653
Symptom status				
Asymptomatic				
Symptomatic	2.204 (1.992–2.438)	<0.001	1.604 (1.401–1.836)	<0.001
TB cases classification				
Positive	1		1	
Negative	3.208 (1.964–5.240)	<0.001	1.086 (0.949–1.242)	0.230
Comorbidities				
No	1		1	
Yes	0.858 (0.778–0.946)	0.002	1.053 (0.818–1.355)	0.690
TB type				
New cases	1		1	
Previously treated cases	1.278 (1.071–1.525)	0.007	0.860 (0.680–1.086)	0.205
Use of FDC				
No	1		1	
Yes	0.189 (0.167–0.215)	<0.001	0.806 (0.666–0.975)	0.026
Co-existing TB				
No	1		1	
Yes	0.763 (0.685–0.851)	<0.001	0.761 (0.642–0.902)	0.002

Multivariable logistic regression analysis revealed that female, other ethnicities, the use of FDC, and co-existing TB were protective against non-use of DAT, with adjusted odds ratios (aORs) of 0.829 (0.716–0.960), 0.555 (0.343–0.898), 0.806 (0.666–0.975) and 0.761 (0.642–0.902), respectively. Compared with Fuzhou, the cities of Putian, Quanzhou, and Ningde had significantly lower odds of non-users of DAT among TB patients, with aORs of 0.024 (0.018–0.030), 0.076 (0.063–0.092), and 0.030 (0.024–0.039), respectively. Conversely, migrant status, symptomatic presentation at diagnosis were identified as independent risk factors for DAT non-use, with aORs of 2.621 (2.144–3.203), 1.604 (1.401–1.836), respectively (Table 2).

### 3.3 Propensity score matching

Propensity score matching (PSM) was performed at a 1:1 ratio using the nearest neighbor method, with a caliper width set at 0.0001. The matching variables included ethnicity, occupational category, current residence (both specific address and city), presenting symptoms, patient detection method, comorbidities, diagnostic classification, treatment category, use of fixed-dose combination (FDC) drugs, and presence of extrapulmonary tuberculosis. A total of 1,372 matched pairs were successfully generated. After matching, no statistically significant differences (*p* > 0.05) were observed in any baseline characteristics between the two groups. Furthermore, all standardized mean differences (SMDs) were below 0.1, confirming adequate balance between the DAT and routine care groups (Table 3).

**Table 3:** Distribution of characteristics between the DAT and routine care groups after propensity score matching.

Characteristics	Total	DAT	Routine	Statistical value	*P*	SMD
Gender				2.852	0.091	0.065
Male	2,211 (80.58)	1,088 (79.30)	1,123 (81.85)			
Female	533 (19.42)	284 (20.70)	249 (18.15)			
Ethnic				0.000	>0.999	<0.001
Han	2,720 (99.13)	1,360 (99.13)	1,360 (99.13)			
Other ethnicities	24 (0.87)	12 (0.87)	12 (0.87)			
Occupation				0.072	>0.999	0.010
Employed in enterprises or public institutions	31 (1.13)	15 (1.09)	16 (1.17)			
Students or children	73 (2.66)	36 (2.62)	37 (2.70)			
Worked in public places or commercial services	46 (1.68)	23 (1.68)	23 (1.68)			
Manual laborers	1793 (65.34)	898 (65.45)	895 (65.23)			
Retired / Unemployed	754 (27.48)	377 (27.48)	377 (27.48)			
Other occupations	47 (1.71)	23 (1.68)	24 (1.75)			
Age (years)				0.109	0.741	0.013
<65	1898 (69.17)	953 (69.46)	945 (68.88)			
≥65	846 (30.83)	419 (30.54)	427 (31.12)			
Specific populations				1.123	0.289	0.040
No	1,689 (61.55)	858 (62.54)	831 (60.57)			
Yes	1,055 (38.45)	514 (37.46)	541 (39.43)			
Residence				0.000	>0.999	<0.001
Local	2,274 (82.87)	1,137 (82.87)	1,137 (82.87)			
Migrant	470 (17.13)	235 (17.13)	235 (17.13)			
City of residence				0.010	>0.999	0.004
Fuzhou	462 (16.84)	231 (16.84)	231 (16.84)			
Putian	189 (6.89)	95 (6.92)	94 (6.85)			
Quanzhou	1858 (67.71)	929 (67.71)	929 (67.71)			
Ningde	235 (8.56)	117 (8.53)	118 (8.60)			
Case finding				0.000	>0.999	<0.001
Active	22 (0.80)	11 (0.80)	11 (0.80)			
Passive	2,722 (99.20)	1,361 (99.20)	1,361 (99.20)			
Symptom status				0.002	0.962	0.002
Asymptomatic	543 (19.79)	271 (19.75)	272 (19.83)			
Symptomatic	2,201 (80.21)	1,101 (80.25)	1,100 (80.17)			
TB cases classification				3.549	0.060	0.072
Positive	2092 (76.24)	1,067 (77.77)	1,025 (74.71)			
Negative	652 (23.76)	305 (22.23)	347 (25.29)			
Comorbidities				0.000	>0.999	<0.001
No	1,638 (59.69)	819 (59.69)	819 (59.69)			
Yes	1,106 (40.31)	553 (40.31)	553 (40.31)			
TB type				0.006	0.939	0.003
New cases	2,559 (93.26)	1,280 (93.29)	1,279 (93.22)			
Previously treated cases	185 (6.74)	92 (6.71)	93 (6.78)			
Use of FDC				0.004	0.953	0.002
No	323 (11.77)	161 (11.73)	162 (11.81)			
Yes	2,421 (88.23)	1,211 (88.27)	1,210 (88.19)			
Co-existing TB				0.000	>0.999	<0.001
No	2,398 (87.39)	1,199 (87.39)	1,199 (87.39)			
Yes	346 (12.61)	173 (12.61)	173 (12.61)			

### 3.4 Differences in treatment adherence between groups

In the overall study population, the sputum smear completion rate at the end of the second treatment month was significantly higher in the DAT group (74.94% [2,909/3882]) than in the routine care group (71.15% [2,664/3744]) (*p* < 0.001). The loss to follow-up rate was 0.67% (26/3882) in the DAT group and 0.61% (23/3744) in the routine care group, with no statistically significant difference between the groups (*p* = 0.762). Among bacteriologically confirmed patients who completed the month-2 smear test, the sputum conversion rate was 91.86% (1930/2101) in the DAT group and 92.68% (1,685/1818) in the routine care group, a difference that was not statistically significant (*p* = 0.337). Within the propensity score-matched cohort, the DAT group continued to demonstrate a significantly higher month-2 sputum smear completion rate (77.33%) compared to the routine care group (71.36%) (*p* < 0.001). The loss to follow-up rate was 0.36% (5/1372) in the DAT group and 0.73% (10/1372) in the routine care group, showing no significant difference (*p* = 0.300). The sputum conversion rate among matched, smear-positive patients was 91.14% (751/824) in the DAT group and 93.58% (670/716) in the routine care group. This difference was not statistically significant (*p* = 0.085) (Table 4).

**Table 4:** Differences in treatment adherence between the DAT and routine care groups in the overall study population and the PSM-matched subgroup.

Treatment adherence	Overall study population			PSM cohort		
	DAT	Routine	*P*	DAT	Routine	*P*
Sputum smear microscopy at the end of the second treatment month			<0.001			<0.001
No	973 (25.06)	1,080 (28.85)		311 (22.67)	393 (28.64)	
Yes	2,909 (74.94)	2,664 (71.15)		1,061 (77.33)	979 (71.36)	
Sputum conversion			0.337			0.085
No	171 (8.14)	133 (7.32)		73 (8.86)	46 (6.42)	
Yes	1930 (91.86)	1,685 (92.68)		751 (91.14)	670 (93.58)	
Loss to follow-up			0.762			0.300
No	3,856 (99.33)	3,721 (99.39)		1,367 (99.64)	1,362 (99.27)	
Yes	26 (0.67)	23 (0.61)		5 (0.36)	10 (0.73)	

## 4 Discussion

Prior research has established that digital adherence technologies (DATs) can achieve treatment adherence outcomes non-inferior to traditional interventions (9, 10). while demonstrating favorable cost-effectiveness (12, 13). Multiple studies have also reported good acceptability and feasibility of DATs (15, 16). The present study found a relatively low utilization rate of DATs (50.9%), which is lower than rates reported in previous studies (11, 14). This discrepancy may be attributed to the different study contexts: our data were derived from real-world implementation, whereas prior evidence largely came from rigorously designed cluster-randomized trials (9) or national DAT promotion programs (12). In our setting, the adoption of DATs was influenced not only by patient preference but also by the willingness of clinicians or nurses at designated TB hospitals to recommend the technology. According to current Chinese TB patient management guidelines, DATs are not formally recommended as a standard management tool. Primary healthcare workers provide routine care as stipulated, while the decision to use DATs is made through consultation between clinicians/nurses at designated hospitals and patients, after which primary healthcare providers are notified. Some hospital-based staff, burdened by heavy workloads, were reluctant to adopt DATs due to the perceived additional effort required to learn and explain the technology to patients (17). Consistent with this, Li et al. found that healthcare workers play a crucial role in improving patient awareness and willingness to use DATs (18).

On the other hand, as a novel adherence intervention, DATs may also raise concerns among patients. For instance, users of the WeChat-based mHealth component worried about personal data privacy and potential complications from information leakage. Users of the smart pillbox expressed concern that the device or its audible reminders could draw unwanted attention from others, thereby exacerbating disease-related stigma and reducing their willingness to use the technology (18). Moving forward, it is recommended to refine TB patient management protocols by incorporating DATs as an optional management strategy. Enhancing training for healthcare workers to improve their knowledge and willingness to promote DATs is also essential. Finally, adherence interventions should be tailored based on patient preferences to facilitate wider and more effective implementation.

We identified several factors associated with a higher likelihood of adopting DATs for adherence support, including female sex, belonging to an ethnic minority group, being treated with fixed-dose combinations (FDCs), and having concurrent extra-pulmonary tuberculosis. Consistent with our findings, a systematic review and a subsequent Chinese study on TB patients with comorbidities both reported superior treatment adherence among females (19, 20). The greater inclination of ethnic minorities to use DATs compared to the Han majority may be linked to their frequent residence in remote rural areas. For these populations, DATs can reduce the need for in-person visits, saving travel time and costs, and facilitate easier communication with managing physicians (15). Furthermore, some evidence suggests that ethnic minority patients may exhibit greater trust in healthcare providers, potentially contributing to higher adherence (21). Patients prescribed FDCs showed a higher DAT uptake rate. As FDCs are specifically designed to simplify regimens and improve adherence (22), patients opting for this treatment may inherently prioritize adherence and thus be more receptive to supplementary tools like DATs. Similarly, patients with more severe disease presentations, such as those with extra-pulmonary involvement, may demonstrate greater trust in medical advice and adherence motivation in their pursuit of recovery.

Notably, willingness to use DATs was higher in other prefectural cities compared to Fuzhou. This may be explained by the centralized care model in Fuzhou, where most patients are treated at provincial-level TB-designated hospitals. In this model, follow-up management is delegated to primary care institutions based on the patient’s residential address. Provincial hospital physicians, burdened by high patient volumes, are less involved in routine health management and may be less likely to recommend DATs. A similarly low DAT uptake among migrant populations can be attributed to this fragmentation of care, where the treating hospital and the managing health authority are in different regions, reducing the clinician’s incentive to promote DATs. Optimizing the implementation workflow by involving the actual care manager in the shared decision-making process with the patient regarding DAT use could potentially improve adoption rates.

Conversely, the presence of symptoms at diagnosis was associated with a lower tendency to use DATs. This may reflect differing levels of health engagement; patients presenting with symptoms might have lower baseline health awareness, which could correlate with generally poorer medication adherence.

Our study found that, both in the real-world cohort and in the propensity score-matched subgroup balanced for potential confounders, patients in the DAT group achieved a significantly higher sputum smear microscopy completion rate at the end of the second treatment month compared to the routine care group. This endpoint serves as a practical indicator of adherence to the treatment regimen (23). Consistent with previous studies, no significant difference was observed in the month-2 sputum conversion rate among bacteriologically confirmed patients between the DAT and routine care groups (9). This may be attributed to several factors. First, sputum conversion is not solely dependent on adherence but is also influenced by drug sensitivity and individual pharmacokinetic variability (24, 25). Second, the overall conversion rates were high in both groups, and the current sample size may be insufficient to detect a statistically significant but modest incremental effect attributable to DATs. The loss to follow-up rates did not differ significantly between the two groups, a result consistent with existing literature (3, 9, 11). Previous research suggests that DATs may be most effective for patients who are already motivated to adhere, whereas they might not fundamentally alter the behavior of those unwilling to adhere, necessitating additional tailored interventions (3). Consequently, future research should focus on developing and evaluating strategies to improve adherence specifically among patients identified as non-adherent through DAT monitoring, with the ultimate goal of enhancing overall treatment success rates.

In summary, the utilization rate of digital adherence technology (DAT) in Fujian Province remains suboptimal, with uptake influenced by individual factors such as patient sex, place of residence, and presence of extrapulmonary tuberculosis. This study provides an empirical basis for future scale-up of electronic adherence interventions. The use of DAT was associated with improved patient adherence to scheduled sputum smear monitoring; however, it did not significantly impact loss to follow-up or sputum conversion rates at the end of the intensive treatment phase. Moving forward, optimizing patient management protocols and DAT implementation workflows is crucial to enhance accessibility and acceptability. Further research should focus on developing effective interventions for patients identified as non-adherent through DAT monitoring, with the aim of maximizing the impact of DATs on both treatment adherence and clinical outcomes.

Our study has several limitations. First, as a retrospective analysis of real-world data, it may be subject to unobserved confounding factors, which could introduce bias and affect the accuracy of our findings. Second, our analysis did not include factors related to healthcare providers. Future research should incorporate comprehensive surveys involving patients, healthcare workers, and program implementers—key stakeholders in DAT implementation—to better assess the influence of each party on the adoption and effectiveness of digital adherence technologies.

## Data Availability

The original contributions presented in the study are included in the article/supplementary material, further inquiries can be directed to the corresponding author.
